# Effect of Low-Intensity Aerobic Training Combined with Blood Flow Restriction on Body Composition, Physical Fitness, and Vascular Responses in Recreational Runners

**DOI:** 10.3390/healthcare10091789

**Published:** 2022-09-16

**Authors:** Hyoung Jean Beak, Wonil Park, Ji Hye Yang, Jooyoung Kim

**Affiliations:** 1 Sports Convergence Institute, Konkuk University, Chungju 27478, Korea; 2 Department of Physical Education, College of Education, Chung-Ang University, Seoul 06974, Korea; 3 Department of Medicine, CHA University, Pocheon 11160, Korea; 4 College of Liberal Arts, Konkuk University, Chungju 27478, Korea

**Keywords:** blood flow restriction, body composition, low-intensity aerobic training, recreational runner, physical fitness, vascular responses

## Abstract

This study investigated the effect of low-intensity aerobic training combined with blood flow restriction (LABFR) on body composition, physical fitness, and vascular functions in recreational runners. The participants were 30 healthy male recreational runners, randomized between the LABFR (*n* = 15) and control (*n* = 15) groups. The LABFR group performed five sets of a repeated pattern of 2 min running at 40% VO_2max_ and 1 min passive rest, while wearing the occlusion cuff belts on the proximal end of the thigh. The frequency was three times a week for the period of eight weeks. The control group performed the identical running protocol without wearing the occlusion cuff belts. At the end of the training, the participants’ body composition (fat mass, body fat, muscle mass, and right and left thigh circumference), physical fitness (power and VO_2max_), and vascular responses (flow-mediated dilation (FMD), brachial ankle pulse wave velocity (baPWV), ankle brachial index (ABI), systolic blood pressure (SBP) and diastolic blood pressure (DBP)) were measured. The results showed a significant time × group interaction effect on muscle mass (F = 53.242, *p* = 0.001, η_p_^2^ = 0.664) and right thigh circumference (F = 4.544, *p* = 0.042, η_p_^2^ = 0.144), but no significant variation in any other factors, including fat mass, body fat, left thigh circumference, FMD, baPWV, ABI, SBP, and DBP (*p* > 0.05). Overall, our results suggested that eight-week LABFR exerted a positive effect on the body composition, especially muscle mass and thigh circumference, of recreational runners.

## 1. Introduction

Recent interest in the application of blood flow restriction (BFR) has focused on the effect of training in general training adaptations during periods of reduced blood flow [1]. In Japan, Dr. Yoshiaki Sato promoted “kaatsu training”, involving “training with added pressure” through which BFR became widely known to the general public [2]. In typical BFR training, a cuff/tourniquet system is used to apply a partial restriction to the arterial inflow in the working musculature during exercise, while the venous outflow is completely restricted [3] so that the consequent blood pooling allows for an increased training effect [4]. Recent evidence has indicated the superiority of the reinforced training stimuli using the combined BFR training compared to the same exercise without BFR training [5].

The BFR method is usually used during low-load resistance exercise, and has been shown to be effective in enhancing long-term hypertrophic and strength responses in both clinical and athletic populations [6]. According to several studies on BFR training, an increase in muscle strength and hypertrophy could be expected from the conventional resistance training only via high-intensity training using an approximately 70–85% load of one-repetition maximum (1 RM), whereas the BFR training exerted a similar level of effect using a 20–50% load of 1 RM [5,6,7]. In fact, BFR training has been shown to offer significant benefits in the change in skeletal muscles in a number of studies, for example, by increasing the local muscle mass, strength, and endurance, despite the training being performed with lower resistance [8]. Moreover, several recent studies have reported a positive effect of BFR training in controlling the arterial compliance compared to the absence of BFR training, as well as stronger advantages in the change in vascular functions through four or more weeks of BFR training compared to conventional resistance training, thus, implicating a potential contribution in cardiovascular health [9].

Meanwhile, changes induced by low-intensity aerobic training combined with BFR (LABFR) have been investigated in recent studies, with the results showing the advantages of BFR training as a single mode of training based on the simultaneous increase in aerobic fitness and muscular strength, in addition to the increased maximum oxygen uptake (VO_2max_), delayed onset of blood lactate accumulation, and enhanced economy of motion [10,11]. For these reasons, LABFR has been applied to individuals with a low level of training or a handicap in certain training, such as those recovering from an injury or those seeking assistive training to add new stimuli to aerobic training, and the reported effects were positive in inducing a variety of physiological changes [12,13,14]. In fact, Abe et al. [15] reported that, although the intensity of slow-walk training with BFR was set to a low level, secretion of growth hormone after acute exercise was increased and, after three weeks, the thigh muscle cross-sectional area and volume were increased by 4–7%. In a recent study by Pinheiro et al. [12], trained cyclists with knee osteoarthritis performed low-intensity aerobic training combined with BFR for nine weeks, in which a positive change was found, not only for aerobic fitness such, as with 20 km cycling time-trial performance and peak oxygen consumption (VO_2peak_), but also for right and left leg maximal strength and the cross-sectional area of the vastus lateralis.

These studies on LABFR involved individuals who were injured or poorly trained. However, in some cases, low-intensity aerobic training is one of the training strategies used by recreational runners. High-intensity interval training can increase catabolic hormones, such as cortisol, which inhibit muscle protein synthesis, reducing muscle hypertrophy and muscle strength development [16,17]. In addition, BFR changes acute physiological stressors, such as local muscle oxygen availability and vascular shear stress, which can lead to adaptations that cannot be easily obtained with traditional training [1]. These advantages can provide positive effects for recreational runners. However, research on the positive effects of LABFR for recreational runners is limited. Thus, this study aimed to investigate the effect of an eight-week protocol of LABFR on body composition, physical fitness, and vascular responses in recreational runners. We hypothesized that regular LABFR training will increase the body composition, physical fitness, and vascular responses in recreational runners.

## 2. Methods

### 2.1. Subjects

Thirty health male recreational runners participated in this study. A recreational runner was defined as an individual who had regularly participated in running three times a week for a minimum of six months. The sample size was estimated by setting the effect size to 0.25 with an alpha level of *p* ≤ 0.05 and statistical power > 0.80. As a result, the sample size required for this study was *n* = 24. Considering the drop-out rate, a total of 30 individuals were recruited. Individuals who received an operation related to a musculoskeletal disorder in the past six months, those with an injury history, and those with a vascular health problem (e.g., hypertension or deep vein thrombosis, or distended varicose veins) or a history of medical treatment or drug administration, were excluded. Prior to the experiment, the participants were given detailed explanations on the purpose, procedures and methods of the experiment, and all participants voluntarily signed an informed consent form. The 30 participants were randomized between the LABFR group (*n* = 15) and the control group (*n* = 15), but 1 from the LABFR group dropped out of the study for a personal reason, leaving 14 individuals in the LABFR group and 15 individuals in the control group at the end of the experiment (Table 1). An independent t-test found no significant differences between groups for the variables of age, height, weight, body mass index (BMI), and body fat (*p* > 0.05). The study procedures and methods, and the consent form, were approved by the university’s Institutional Review Board. The study was conducted between April 2021 and January 2022.

### 2.2. Low-Intensity Aerobic Training Combined with Blood Flow Restriction

**Figure 1:**
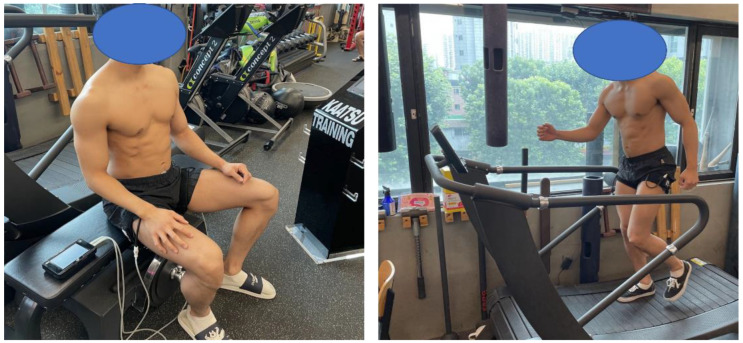
Occlusion cuff belts used in this study for blood flow restriction.

Participants visited a laboratory with BFR equipment to perform training. Here, VO_2max_ was measured for LABFR. Before the VO_2max_ measurement, the participants attended an education session on relevant procedures and precautions. Afterwards, each participant performed a warm-up on the treadmill, followed by exercise at 1.7 mph with 10% slope. At every 3 min interval, the speed was increased by 0.8–0.9 mph, and the slope was increased by 2% as part of the Bruce protocol to measure the VO_2max_. In the measurements, gas analyzers (Quark b, Cosmed, Rome, Italy) were concurrently applied, after adjustments for the daily volume, humidity, and temperature. The criteria of VO_2max_ were (i) a change in VO_2_ ≤ 2 mL·kg^−1^·min^−1^; (ii) respiratory exchange ratio ≥ 1.1; (iii) age-predicted maximal heart rate ≥ 85%. Before performing LABFR, 10 min of light jogging was performed as a warm-up. Afterwards, each participant wore an occlusion cuff belt (KAATSU NANO, KAATSU JAPAN, Tokyo, Japan) at the proximal end of both thighs (Figure 1). For LABFR, each participant repeated a pattern of 2 min running on a treadmill (HERA-9000, Health-One, Goyang, Republic of Korea) with the exercise intensity set to 40% VO_2max_, followed by a 1 min passive rest, performing a total of five sets. The total time taken for exercise was 15 min. The pressure range during LABFR was set to 160–240 mmHg, because the use of LABFR with higher occlusion pressures (≥130 mmHg) has been shown to improve both the aerobic fitness and aerobic performance in young adults [18]. Participants performed LABFR three times per week for a period of eight weeks. They were instructed to take sufficient recovery time after training, and were also asked to avoid consuming caffeine-containing foods and beverages for 24 h before training. The LABFR protocol in this study was designed with reference to previous studies [2,18]. The LABFR protocol used in this study was registered at https://www.protocols.io/ (accessed on 20 July 2022). The control group performed the same protocol as the LABFR group without wearing the occlusion cuff belts for blood flow restriction.

### 2.3. Body Composition

The body composition (fat mass, body fat, and muscle mass) of the participants was measured using a bioelectrical impedance analysis device (InBody-270, Biospace, Seoul, Republic of Korea) [19]. For accurate measurements, the participants were prohibited from performing excessive physical activity or exercise on the day prior to the day of measurement. They were also advised not to drink caffeinated or alcoholic beverages with a potential effect on moisture imbalance, although they were allowed to drink an adequate quantity of water. After a minimum of 8 h of fasting, the measurements were taken during the morning hours. The thigh circumference was also measured to examine the changes in muscle hypertrophy [20]. For this, while the participant’s leg was in a natural extension state, a spot 18 cm from the knee joint was marked with ink and the circumference around the spot was measured using a fiberglass tape (Gulick II, Country Technology Inc., Gays Mills, WI, USA).

### 2.4. Physical Fitness

The physical fitness was measured and subdivided into power and cardiorespiratory fitness. Power was measured using the vertical jump. For this, a jump-MD (TKK-5406, TAKEI, Niigata, Japan) was used. While standing on a mat with the feet aligned with each respective shoulder, the participant made as high a vertical jump as possible through the instant flexion and extension of the knees at the investigator’s signal. The measurements were taken twice, and the higher of the two values was recorded. The participant was cautioned against jumping through rebounding. For cardiorespiratory fitness, the VO_2max_ was measured. The measurement procedure for VO_2_max has already been described in detail in Section 2.2.

### 2.5. Vascular Responses

Flow-mediated dilation (FMD) is a method of assessing the endothelial functions based on the level of expansion of the brachial artery in response to the nitric oxide produced by vascular endothelial cells. To measure the FMD, an ECG-guided high-resolution B-mode ultrasound system (UNEX-EF-38G, UNEX Corp., Nagoya, Japan) was used to measure the baseline diameter of the brachial artery at rest, and the maximum diameter after applying shear stress to express the change in brachial arterial diameter as a ratio was determined [21]. The respective equation for FMD is as follows: FMD = [(maximal diameter–baseline diameter)/baseline diameter × 100]. The brachial ankle pulse wave velocity (baPWV) of each participant in the supine position was measured using a non-invasive vascular screening device (VP-1000 Plus, Omron, Kyoto, Japan) after at least 5 min of rest. The sampling time for one pulse wave recording was 10 s. For each participant, two consecutive measurements were taken and the mean of two values was used in the analysis. The factors that determine a pulse wave are time difference (ΔT), distance between two measured spots (L), and participant’s height (cm). The pulse wave (L/ΔT, cm/s) for an arterial segment was automatically calculated by the device [22]. Ankle brachial index (ABI) was measured using the same device as in the baPWV measurements. The cuff was applied to the limbs of each participant in the supine position, after at least 10 min of rest. The systolic blood pressure (SBP) was measured at the left and right brachial artery and the left and right posterior tibial artery, and by dividing the highest posterior tibial arterial pressure by the highest brachial arterial pressure, the ratio for ABI was obtained [23]. The respective equation for ABI was as follows: ABI = (highest left and right ankle systolic blood pressure)/(highest left and right arm systolic blood pressure).

### 2.6. Statistical Analysis

All data in this study were presented as the mean and standard deviation. The normality and homogeneity were tested using the Kolmogorov–Smirnov and Levene’s test, respectively. To analyze the time × group interaction effect, a repeated measure ANOVA was applied, and Bonferroni procedures were used for post hoc comparison. The partial eta squared (η_p_^2^) was used to determine the effect size in the mixed ANOVA. All analyses were performed using the SPSS software (SPSS Statistics 25.0, IBM, Armonk, NY, USA). The level of significance was set to 0.05.

## 3. Results

Table 2 presents the changes in body composition after performing the eight-week LABFR protocol. All body composition variables were normally distributed, as assessed using the Kolmogorov–Smirnov test (*p* > 0.05), except for pre- and post-body fat and pre- and post-right thigh circumference (*p* < 0.05). Levene’s test showed no significant difference in variances for all body composition variables (*p* > 0.05). The results indicated no significant time × group interaction effect on fat mass (F = 0.265, *p* = 0.611, η_p_^2^ = 0.010) and body fat (F = 0.490, *p* = 0.490; η_p_^2^= 0.018). In contrast, significant time × group interactions effects were found on muscle mass (F = 53.242, *p* = 0.001, η_p_^2^ = 0.664) and right thigh circumference (F = 4.544, *p* = 0.042, η_p_^2^ = 0.144). Compared to the control group, the LABFR group exhibited an increase in muscle mass and right thigh circumference, whereas no interaction effect was found in left thigh circumference (F = 3.171, *p* = 0.086, η_p_^2^ = 0.105).

Table 3 presents the changes in physical fitness after the eight-week LABFR protocol. All physical fitness variables were normally distributed, as assessed using the Kolmogorov–Smirnov test (*p* > 0.05). Levene’s test showed that pre- and post-power were significantly different variances (*p* < 0.05). The results indicated no significant time × group interaction effect on either power (F = 0.624, *p* = 0.437, η_p_^2^ = 0.023) or VO_2max_ (F = 0.258, *p* = 0.616, η_p_^2^ = 0.009). Lastly, Table 4 presents the changes in vascular responses after the eight-week LABFR protocol. All vascular responses variables were normally distributed, as assessed using Kolmogorov–Smirnov test (*p* > 0.05). Levene’s test showed no significant difference in variances for all vascular response variables (*p* > 0.05). The results showed no significant time × group interaction effect on FMD (F = 0.042, *p* = 0.840, η_p_^2^ = 0.002) or any other factors, as follows: baPWV (F = 0.073, *p* = 0.789, η_p_^2^ = 0.003) and ABI (F = 0.188, *p* = 0.668, η_p_^2^ = 0.007), SBP (F = 0.039, *p* = 0.845, η_p_^2^ = 0.001), and DBP (F = 2.354, *p* = 0.137, η_p_^2^ = 0.080).

## 4. Discussion

The purpose of this study was to investigate the effect of an eight-week LABFR protocol on the body composition, physical fitness, and vascular responses in recreational runners. The results indicated a higher level of increase in muscle mass and thigh circumference in the LABFR group compared to the control group, which agrees with the results of a number of previous studies. Abe et al. [13] and Kim et al. [14] had reported a positive effect of LABFR on the cross-sectional area and volume of thigh and quadriceps muscles. In Abe et al. [13], when young adults who did not regularly participate in aerobic training performed LABFR for eight weeks, the cross-sectional area and volume of the thigh and quadriceps muscles showed a significant increase compared to the control group. In Kim et al. [14], healthy undergraduates performed LABFR for six weeks, as a result of which the leg muscle mass significantly increased, and the knee flexion muscle strength increased to a similar level as in vigorous-intensity cycling.

A number of studies suggested that the BFR training may promote muscle growth by activating the intracellular signaling pathways for muscle protein synthesis, such as the mTOR pathway, through the creation of a hypoxic environment inside the muscle [24], and by altering the genetic regulation of muscle satellite cells [25]. Another study claimed that the muscle hypertrophy induced through BFR could include increased mechanical tension and metabolic stress and muscle growth as a result of autocrine and/or paracrine actions [26]. However, it should be noted that the BFR-mediated mechanisms related to increased muscle mass or hypertrophy had mainly been based on a resistance training model. Abe et al. [13] and Kim et al. [14], likewise, could not clearly identify the potential mechanism of LABFR, but suggested only a conjectured mechanism based on resistance training models used in previous studies despite the reported positive effect of LABFR. Thus, further studies should investigate the potential mechanisms involved in the post-LABFR changes. An interesting finding in this study was the lack of change in power despite the increased muscle mass and thigh circumference. While certain previous studies have reported the possibility of a positive relation between increasing the muscle mass and improving the muscle force, others reported a very weak or lack of a relationship [27,28]. It is also noteworthy that only the right thigh circumference showed a significant increase after LABFR in this study. The reason for this change is not clear and should be investigated in future research.

Likewise, in this study, we hypothesized that there would be a significant change in physical fitness and vascular responses based on several previous studies suggesting a potential additional training effect from ischemia–reperfusion caused by cuff pressure on oxidative metabolism and angiogenesis [29,30]. However, the eight-week LABFR protocol had not exerted any effect on either physical fitness measure, including VO_2max_ or vascular responses, such as FMD, baPWV, ABI, SBP, and DBP, in contrast to recent reports. The study of Karabulut et al. [31], in which healthy and active young male adults performed six-weeks of LABFR training on a treadmill, reported that the VO_2max_ was higher in the LABFR group than in the control group. In Pinheiro et al. [12], where trained cyclists performed a 9-week course of LABFR training, it was also reported that LABFR contributed to improving the VO_2peak_. Likewise, in de Oliveira et al. [32], four-week LABFR training could improve the onset of blood lactate accumulation in active males.

However, the participants in Karabulut et al. [31] and de Oliveira et al. [32] were males from the general population, and did not regularly participate in endurance training programs, while the participant in Pinheiro et al. [12] was a trained cyclist with knee osteoarthritis, which deviated from the present investigation. Moreover, Pinheiro et al. [12] was a case report on one trained cyclist. The difference in subject characteristics is likely to have had an influence on vascular responses. The participants in this study were recreational runners without any physical problems, who had been performing regular running for a long period of time before participating in this study, and it is likely to have been difficult to induce a significant change in vascular responses.

This study has a few limitations. First, the subjects of this study included only recreational runners who regularly participated in running. Therefore, the effect of LABFR on individuals regularly participating in other types of exercise remains unknown. In a follow-up study, the effect of LABFR should be investigated in different exercise models. Second, a bioelectrical impedance analysis device and a fiberglass tape were used to measure the changes in muscle mass and thigh circumference. In addition, power was measured through vertical jump. A greater variety of instruments could not be used due to the limitations of laboratory conditions. In the future, the changes in skeletal muscles should be monitored using a magnetic resonance imager or an isokinetic dynamometer to extend the scope of the instruments.

## 5. Conclusions

To conclude, our results indicated that the eight-week LABFR protocol had a potential positive effect on the body composition, especially muscle mass and thigh circumference, in recreational runners. These findings suggest that LABFR can be a useful training strategy for recreational runners seeking muscle-related changes. However, the lack of significant changes in indicators, such as VO_2max_ or vascular responses that are more closely associated with the runner, raises a question regarding the practical effectiveness of LABFR, while implying that care should be taken in result interpretations. The fact that only a few studies similar to the present investigation have been conducted, as well as the limitations of the present investigation, should also be considered. In the future, a greater number of studies investigating the effect of LABFR in recreational runners using a reliable design and more varied indicators should be conducted.

## Figures and Tables

**Table 1:** Physical characteristics of the subjects.

	LABFR (*n* = 14)	CON (*n* = 15)
Age (years)	30.21 ± 4.93	29.67 ± 3.06
Height (cm)	175.07 ± 8.46	172.27 ± 5.66
Weight (kg)	75.23 ± 9.08	73.59 ± 10.68
BMI (kg/m^2^)	24.44 ± 2.32	24.63 ± 2.77
Body fat (%)	12.54 ± 4.27	15.33 ± 5.05

Data are presented as mean ± standard deviation (SD); BMI, body mass index; LABFR, low-intensity aerobic training combined with blood flow restriction group; CON, control group.

**Table 2:** Change in body composition after low-intensity aerobic training combined with BFR.

	Group	Pre	Post	F	*p*	η_p_^2^
Fat mass (kg)	BFR (*n* = 14)	12.54 ± 4.27	11.56 ± 3.09	0.265	0.611	0.010
	CON (*n* = 15)	15.33 ± 5.05	14.52 ± 4.77			
Body fat (%)	BFR (*n* = 14)	16.90 ± 6.42	15.82 ± 6.63	0.490	0.490	0.018
	CON (*n* = 15)	20.92 ± 6.88	20.18 ± 6.76			
Muscle mass (kg)	BFR (*n* = 14)	35.59 ± 6.08	36.06 ± 5.91	53.242	0.001 ***	0.664
	CON (*n* = 15)	32.98 ± 6.13	32.46 ± 6.08			
Thigh circumference (right, cm)	BFR (*n* = 14)	56.42 ± 4.23	57.10 ± 4.32	4.544	0.042 *	0.144
	CON (*n* = 15)	55.26 ± 5.43	55.36 ± 5.54			
Thigh circumference (left, cm)	BFR (*n* = 14)	56.07 ± 3.94	57.10 ± 4.27	3.171	0.086	0.105
	CON (*n* = 15)	55.00 ± 5.53	55.46 ± 5.44			

Data are presented as mean ± standard deviation (SD); * *p* < 0.05, *** *p* < 0.001; BFR, blood flow restriction; LABFR, low-intensity aerobic training combined with blood flow restriction group; CON, control group.

**Table 3:** Change in physical fitness after low-intensity aerobic training combined with BFR.

	Group	Pre	Post	F	*p*	η_p_^2^
Power (cm)	BFR (*n* = 14)	57.57 ± 8.21	62.71 ± 8.38	0.624	0.437	0.023
	CON (*n* = 15)	48.93 ± 14.16	53.06 ± 12.10			
VO_2max_ (mL/kg/min)	BFR (*n* = 14)	48.59 ± 8.56	51.74 ± 9.45	0.258	0.616	0.009
	CON (*n* = 15)	44.01 ± 6.80	46.48 ± 6.58			

Data are presented as mean ± standard deviation (SD); BFR, blood flow restriction; LABFR, low-intensity aerobic training combined with blood flow restriction group; CON, control group; VO_2max_, maximum oxygen uptake.

**Table 4:** Change in vascular responses after low-intensity aerobic training combined with BFR.

	Group	Pre	Post	F	*p*	η_p_^2^
FMD (%)	BFR (*n* = 14)	7.27 ± 2.96	7.39 ± 2.02	0.042	0.840	0.002
	CON (*n* = 15)	6.73 ± 1.88	7.01 ± 1.95			
baPWV (cm/s)	BFR (*n* = 14)	1216.07 ± 139.47	1141.89 ± 154.45	0.073	0.789	0.003
	CON (*n* = 15)	1179.93 ± 133.21	1110.76 ± 139.25			
ABI	BFR (*n* = 14)	1.11 ± 0.071	1.01 ± 0.115	0.188	0.668	0.007
	CON (*n* = 15)	1.09 ± 0.074	1.00 ± 0.082			
SBP (mmHg)	BFR (*n* = 14)	126.71 ± 7.31	118.35 ± 7.93	0.039	0.845	0.001
	CON (*n* = 15)	125.46 ± 9.50	117.53 ± 6.34			
DBP (mmHg)	BFR (*n* = 14)	78.00 ± 9.79	67.21 ± 5.82	2.354	0.137	0.080
	CON (*n* = 15)	76.13 ± 9.14	69.93 ± 8.22			

Data are presented as mean ± standard deviation (SD); BFR, blood flow restriction; LABFR, low-intensity aerobic training combined with blood flow restriction group; CON, control group; ABI, ankle brachial index; baPWV, brachial ankle pulse wave velocity; FMD, flow-mediated dilation; SBP, systolic blood pressure; DBP, diastolic blood pressure.

## Data Availability

The data are not publicly available due to privacy or ethical reasons.
